# [(1*R*)-3-Benzoyl-1,7,7-trimethyl­bicyclo­[2.2.1]heptan-2-onato-κ^2^ *O*,*O*′]chlorido(η^6^-*p*-cymene)ruthenium(II)

**DOI:** 10.1107/S1600536810005015

**Published:** 2010-02-13

**Authors:** Mohamed Anouar Harrad, Pedro Valerga, M. Carmen Puerta, Mustapha Ait Ali, Larbi El Firdoussi, Abdellah Karim

**Affiliations:** a Laboratoire de Chimie de Coordination, Faculté des Sciences-Semlalia, BP 2390, 40001 Marrakech, Morocco; b Departamento de Ciencia de los Materiales e Ingeniería Metalúrgica, Facultad de Ciencias, Campus Universitario del Río San Pedro, Puerto Real 11510, Spain

## Abstract

The asymmetric unit of the title compound, [RuCl(C_10_H_14_)(C_17_H_19_O_2_)], contains two diastereomers. In both, the Ru^II^ ion has a tetra­hedral coordination, formed by two O atoms of the camphor-derived ligand and the *p*-cymene and Cl ligands. In the crystal structure, weak inter­molecular C—H⋯Cl inter­actions link the mol­ecules into columns propagated along [010].

## Related literature

For camphor-derived 1,3-diketonato ligands, see: Togni (1990 ▶); Togni *et al.* (1993 ▶). For applications of their transition metal complexes as therapeutic drugs, see: Guo & Sadler (1999 ▶). For related structures, see: Ait Ali *et al.* (2006 ▶); Spannenberg *et al.* (2002 ▶).
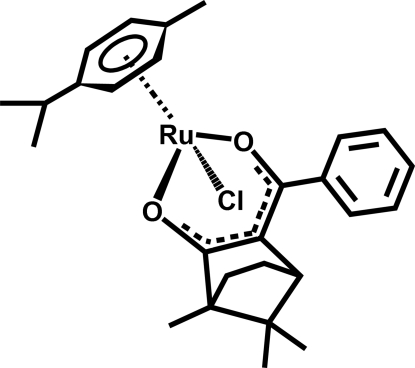


## Experimental

### 

#### Crystal data

[RuCl(C_10_H_14_)(C_17_H_19_O_2_)]
*M* *_r_* = 526.05
Triclinic, 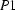

*a* = 9.833 (2) Å
*b* = 10.572 (2) Å
*c* = 12.785 (3) Å
α = 108.13 (3)°
β = 97.62 (3)°
γ = 102.54 (3)°
*V* = 1203.9 (6) Å^3^
*Z* = 2
Mo *K*α radiation
μ = 0.78 mm^−1^
*T* = 100 K
0.56 × 0.31 × 0.24 mm

#### Data collection

Bruker SMART APEX diffractometer
Absorption correction: multi-scan (*SADABS*; Sheldrick, 2004 ▶) *T*_min_ = 0.762, *T*_max_ = 0.961
10110 measured reflections
7861 independent reflections
7619 reflections with *I* > 2σ(*I*)
*R*_int_ = 0.020

#### Refinement

*R*[*F* ^2^ > 2σ(*F* ^2^)] = 0.031
*wR*(*F* ^2^) = 0.087
*S* = 1.06
7861 reflections
547 parameters
3 restraints
H-atom parameters constrained
Δρ_max_ = 0.95 e Å^−3^
Δρ_min_ = −1.34 e Å^−3^
Absolute structure: Flack (1983 ▶), 2564 Friedel pairs
Flack parameter: 0.03 (4)

### 

Data collection: *SMART* (Bruker, 2001 ▶); cell refinement: *SAINT* (Bruker, 2001 ▶); data reduction: *SAINT*; program(s) used to solve structure: *SHELXTL* (Sheldrick, 2008 ▶); program(s) used to refine structure: *SHELXTL*; molecular graphics: *ORTEP-3* (Farrugia, 1997 ▶); software used to prepare material for publication: *SHELXTL*.

## Supplementary Material

Crystal structure: contains datablocks global, I. DOI: 10.1107/S1600536810005015/cv2696sup1.cif

Structure factors: contains datablocks I. DOI: 10.1107/S1600536810005015/cv2696Isup2.hkl

Additional supplementary materials:  crystallographic information; 3D view; checkCIF report

## Figures and Tables

**Table 1:** Hydrogen-bond geometry (Å, °)

*D*—H⋯*A*	*D*—H	H⋯*A*	*D*⋯*A*	*D*—H⋯*A*
C43—H43⋯Cl1	0.95	2.69	3.605 (7)	162
C14—H14⋯Cl2	0.95	2.78	3.731 (7)	178

**Fig. 1.**
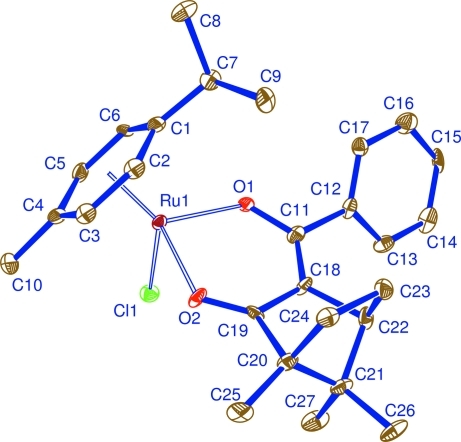
First independent molecule of (I) with the atomic numbering and 50% probability displacement ellipsoids.

**Fig. 2.**
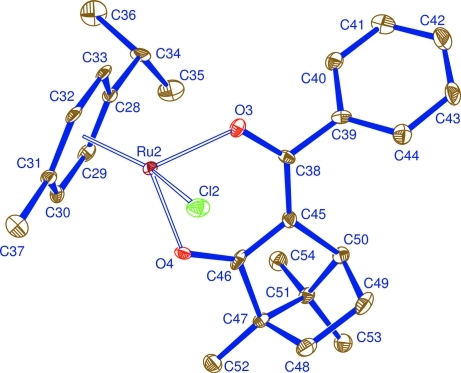
Second independent molecule of (I) with the atomic numbering and 50% probability displacement ellipsoids.

## supplementary crystallographic information

### Comment

Camphor-derived 1,3-diketonato ligands are a potentially attractive class of ligands in organometallic development, because these compounds are readily synthesized, easily varied (Togni *et al.*, 1990, 1993) and some of their corresponding transition metal complexes can be used as therapeutic drugs (Guo *et al.*, 1999). The crystallographic study of these compounds is of great interest in view of search for structure–activity relationships. This paper is a continuation of our X-ray crystal structure studies on rhodium and ruthenium complexes incorporating camphor-derived 1,3-diketonato ligands (Spannenberg *et al.*, 2002; Ait Ali *et al.*, 2006).

The title complex (I) was synthesized by addition of [RuCl_2_(*p*-cymene)]_2_ to a mixture of (1*R*)-3-Benzoyl-1,7,7-trimethyl-bicyclo[2.2.1]heptan-2-one, also named (1*R*)-3-benzoyl-camphor, and Na_2_CO_3_ in anhydrous THF. The neutral complex [RuCl(η^6^-*p*-cymene){κ^2^*O*,*O*'-(1*R*)-3-benzoyl- camphor}] was obtained. The metal centre shows tetrahedral coordination formed by two O atoms of the camphor-derived ligand, and the *p*-cymene and Cl ligands. Two independent molecules in the unit cell have opposite configuration at the metallic centre (R and S), but both of them keep the initial configuration at the two chiral carbon atoms in the (1*R*)-3-benzoyl-camphor free ligand being, consequently, diasteomers and only partially enantiomers.

In the crystal structure, weak intermolecular C—H···Cl interactions (Table 2) link the molecules into columns propagated in direction [010].

### Experimental

A solution of [RuCl_2_(*p*-cymene)]_2_ (100 mg, 0.163 mmol) in 10 ml of THF was added to a suspension of (1*R*)-(+)-3-benzoyl-camphor (83.13 mg, 0.326 mmol) and Na_2_CO_3_ (103 mg, 0.978 mmol) in 10 ml of THF. The mixture was stirred for 3 h at room temperature. It was then evaporated to dryness under reduced pressure. The residue was extracted with CH_2_Cl_2_. The recovered filtrate was evaporated to dryness and lead to an orange solid with an output of 90%. The solid was recrystallized in diethylether. ^1^H NMR (δ, p.p.m.): 0.76 (s, 6H), 0.9 (s, 1H), 1.22 (d, 6H), 1.23–1.3 (m, 4H), 2.2 (s, 3H), 2.5 (d, 1H), 2.8 (m, 1H), 5.11 (d, 2H), 5.34 (d, 2H), 7.18–7.47 (m, 5H, Ar). ^13^C{^1^H} NMR (δ, p.p.m.): 9.8, 10.23, 17.93, 18.4, 19.28, 20.1, 20.4, 22.74, 27.47, 28.59,(28.71), 30.80, 31.09, (31.17), 50.20, 52.23, 58.35, 78.88, 82.74, 98.82, 99.16, 112.71, 113.68, 128.7, 129.16, 140.03, 174.11, 200.48, 201.01

### Refinement

All H atoms were positioned geometrically (C—H = 0.95–0.99Å) and treated as riding, with U_iso_(H) = 1.2U_eq_(C).

### Crystal data

**Table:**

[RuCl(C_10_H_14_)(C_17_H_19_O_2_)]	*Z* = 2
*M**_r_* = 526.05	*F*(000) = 544
Triclinic, *P*1	*D*_x_ = 1.451 Mg m^−^^3^
Hall symbol: P 1	Mo *K*α radiation, λ = 0.71073 Å
*a* = 9.833 (2) Å	Cell parameters from 7193 reflections
*b* = 10.572 (2) Å	θ = 2.5–27.6°
*c* = 12.785 (3) Å	µ = 0.78 mm^−^^1^
α = 108.13 (3)°	*T* = 100 K
β = 97.62 (3)°	Prism, orange
γ = 102.54 (3)°	0.56 × 0.31 × 0.24 mm
*V* = 1203.9 (6) Å^3^	

### Data collection

**Table:**

Bruker SMART APEX diffractometer	7861 independent reflections
Radiation source: fine-focus sealed tube	7619 reflections with *I* > 2σ(*I*)
graphite	*R*_int_ = 0.020
1700 ω scan frames, 0.3 deg, 10 sec	θ_max_ = 27.6°, θ_min_ = 1.7°
Absorption correction: multi-scan (*SADABS*; Sheldrick, 2004)	*h* = −9→12
*T*_min_ = 0.762, *T*_max_ = 0.961	*k* = −13→13
10110 measured reflections	*l* = −16→16

### Refinement

**Table:**

Refinement on *F*^2^	0 constraints
Least-squares matrix: full	H-atom parameters constrained
*R*[*F*^2^ > 2σ(*F*^2^)] = 0.031	*w* = 1/[σ^2^(*F*_o_^2^) + (0.0375*P*)^2^ + 2.2953*P*] where *P* = (*F*_o_^2^ + 2*F*_c_^2^)/3
*wR*(*F*^2^) = 0.087	(Δ/σ)_max_ = 0.001
*S* = 1.06	Δρ_max_ = 0.95 e Å^−^^3^
7861 reflections	Δρ_min_ = −1.34 e Å^−^^3^
547 parameters	Absolute structure: Flack (1983), 2564 Friedel pairs
3 restraints	Flack parameter: 0.03 (4)

### Special details

**Table:**

Experimental. Bruker SMART APEX 3-circle diffractometer with CCD area detector, sealed X-ray tube, graphite monochromator. A hemisphere of the reciprocal space up to theta(max) = 27.56 deg was measured by omega scan frames with delta(omega) = 0.30 deg and 10 sec per frame, 1700 frames were recorded using program SMART (Bruker). Frame data evaluation and integration were done with program SAINT+(Bruker); Lattice parameters by least-squares refinement of the geometric parameters of the strongest reflections with program SAINT + (Bruker). Correction for absorption and crystal decay (insignificant) were applied by semi-empirical method from equivalents using program SADABS (G.M. Sheldrick, version of 2001, Univ. of Goettingen, Germany). Data reduction was done with program XPREP (BRUKER).
Geometry. All esds (except the esd in the dihedral angle between two l.s. planes) are estimated using the full covariance matrix. The cell esds are taken into account individually in the estimation of esds in distances, angles and torsion angles; correlations between esds in cell parameters are only used when they are defined by crystal symmetry. An approximate (isotropic) treatment of cell esds is used for estimating esds involving l.s. planes.
Refinement. Refinement of *F*^2^ against unique set of reflections. The weighted *R*-factor wR and goodness of fit *S* are based on *F*^2^, conventional *R*-factors *R* are based on *F*, with *F* set to zero for negative *F*^2^. The threshold expression of *F*^2^ > σ(*F*^2^) is used only for calculating *R*-factors(gt) etc. and is not relevant to the choice of reflections for refinement. *R*-factors based on *F*^2^ are statistically about twice as large as those based on *F*, and *R*- factors based on ALL data will be even larger.

### Fractional atomic coordinates and isotropic or equivalent isotropic displacement parameters (Å^2^)

**Table:**

	*x*	*y*	*z*	*U*_iso_*/*U*_eq_	
Ru1	0.14364 (3)	0.83889 (3)	0.44793 (2)	0.01034 (11)	
Cl1	0.3688 (2)	0.7926 (2)	0.49401 (15)	0.0171 (4)	
O1	0.0772 (6)	0.7426 (6)	0.5592 (4)	0.0124 (11)	
O2	0.0867 (6)	0.6470 (5)	0.3196 (4)	0.0165 (12)	
C1	−0.0099 (9)	0.9619 (9)	0.4831 (7)	0.0163 (12)	
C2	−0.0163 (10)	0.9068 (9)	0.3639 (6)	0.0167 (11)	
H2	−0.1058	0.8554	0.3142	0.020*	
C3	0.1045 (9)	0.9267 (8)	0.3198 (6)	0.0167 (11)	
H3	0.0959	0.8882	0.2407	0.020*	
C4	0.2426 (9)	1.0043 (9)	0.3907 (7)	0.0163 (12)	
C5	0.2486 (9)	1.0584 (8)	0.5096 (7)	0.0169 (13)	
H5	0.3381	1.1089	0.5596	0.020*	
C6	0.1293 (8)	1.0392 (8)	0.5525 (6)	0.0123 (12)	
H6	0.1383	1.0785	0.6316	0.015*	
C7	−0.1356 (8)	0.9335 (9)	0.5332 (7)	0.0178 (17)	
H7	−0.1003	0.9336	0.6103	0.021*	
C8	−0.2081 (11)	1.0542 (10)	0.5469 (9)	0.033 (2)	
H8A	−0.2423	1.0576	0.4725	0.050*	
H8B	−0.1386	1.1423	0.5936	0.050*	
H8C	−0.2889	1.0382	0.5830	0.050*	
C9	−0.2507 (9)	0.7956 (9)	0.4684 (8)	0.0239 (18)	
H9A	−0.3281	0.7874	0.5089	0.036*	
H9B	−0.2084	0.7184	0.4623	0.036*	
H9C	−0.2886	0.7933	0.3928	0.036*	
C10	0.3731 (9)	1.0207 (9)	0.3443 (8)	0.0237 (18)	
H10A	0.4118	1.1182	0.3540	0.036*	
H10B	0.3495	0.9643	0.2640	0.036*	
H10C	0.4443	0.9904	0.3844	0.036*	
C11	0.0264 (8)	0.6110 (8)	0.5354 (6)	0.0142 (15)	
C12	−0.0215 (8)	0.5737 (8)	0.6320 (6)	0.0155 (15)	
C13	0.0235 (8)	0.4731 (8)	0.6649 (6)	0.0174 (15)	
H13	0.0810	0.4240	0.6236	0.021*	
C14	−0.0142 (8)	0.4439 (8)	0.7564 (6)	0.0240 (16)	
H14	0.0166	0.3749	0.7783	0.029*	
C15	−0.0977 (8)	0.5162 (8)	0.8164 (6)	0.0216 (15)	
H15	−0.1226	0.4974	0.8804	0.026*	
C16	−0.1451 (9)	0.6153 (9)	0.7845 (6)	0.0241 (17)	
H16	−0.2046	0.6623	0.8248	0.029*	
C17	−0.1043 (8)	0.6456 (8)	0.6919 (6)	0.0152 (15)	
H17	−0.1338	0.7156	0.6706	0.018*	
C18	0.0064 (8)	0.5095 (8)	0.4311 (6)	0.0140 (14)	
C19	0.0341 (8)	0.5356 (8)	0.3317 (6)	0.0126 (14)	
C20	−0.0353 (6)	0.4018 (5)	0.2333 (4)	0.0164 (10)	
C21	−0.0127 (6)	0.2937 (5)	0.2883 (4)	0.0183 (10)	
C22	−0.0807 (6)	0.3561 (5)	0.3890 (4)	0.0172 (10)	
H22	−0.0800	0.3109	0.4471	0.021*	
C23	−0.2285 (6)	0.3514 (5)	0.3298 (4)	0.0192 (10)	
H23A	−0.2683	0.4205	0.3786	0.023*	
H23B	−0.2957	0.2583	0.3075	0.023*	
C24	−0.1973 (5)	0.3875 (5)	0.2251 (4)	0.0175 (10)	
H24A	−0.2192	0.4753	0.2274	0.021*	
H24B	−0.2542	0.3129	0.1546	0.021*	
C25	0.0181 (9)	0.3967 (9)	0.1269 (6)	0.0229 (18)	
H25A	0.0018	0.4739	0.1048	0.034*	
H25B	−0.0334	0.3090	0.0664	0.034*	
H25C	0.1204	0.4039	0.1405	0.034*	
C26	−0.0953 (9)	0.1438 (8)	0.2157 (7)	0.0236 (17)	
H26A	−0.0790	0.0823	0.2569	0.035*	
H26B	−0.0620	0.1168	0.1452	0.035*	
H26C	−0.1974	0.1366	0.1989	0.035*	
C27	0.1446 (6)	0.3002 (6)	0.3233 (5)	0.0255 (12)	
H27A	0.1989	0.3960	0.3676	0.038*	
H27B	0.1824	0.2670	0.2558	0.038*	
H27C	0.1529	0.2417	0.3688	0.038*	
Ru2	0.32581 (3)	0.12766 (3)	0.89620 (2)	0.00965 (11)	
Cl2	0.1007 (2)	0.1732 (2)	0.84692 (15)	0.0175 (4)	
O3	0.3958 (6)	0.2229 (5)	0.7863 (4)	0.0128 (11)	
O4	0.3740 (6)	0.3211 (6)	1.0232 (4)	0.0120 (11)	
C28	0.4792 (8)	0.0063 (8)	0.8589 (6)	0.0120 (10)	
C29	0.4905 (9)	0.0603 (8)	0.9753 (6)	0.0140 (12)	
H29	0.5809	0.1113	1.0237	0.017*	
C30	0.3639 (8)	0.0387 (8)	1.0228 (6)	0.0134 (12)	
H30	0.3733	0.0777	1.1020	0.016*	
C31	0.2307 (8)	−0.0369 (8)	0.9555 (6)	0.0120 (10)	
C32	0.2195 (8)	−0.0920 (8)	0.8365 (6)	0.0127 (11)	
H32	0.1285	−0.1421	0.7887	0.015*	
C33	0.3411 (9)	−0.0737 (8)	0.7879 (6)	0.0127 (11)	
H33	0.3314	−0.1141	0.7088	0.015*	
C34	0.6049 (8)	0.0281 (9)	0.8037 (7)	0.0173 (16)	
H34	0.5684	0.0286	0.7271	0.021*	
C35	0.7144 (10)	0.1657 (10)	0.8690 (8)	0.030 (2)	
H35A	0.6654	0.2367	0.8971	0.045*	
H35B	0.7740	0.1919	0.8194	0.045*	
H35C	0.7745	0.1574	0.9328	0.045*	
C36	0.6654 (10)	−0.0953 (11)	0.7886 (8)	0.030 (2)	
H36A	0.6936	−0.1035	0.8621	0.046*	
H36B	0.7489	−0.0823	0.7549	0.046*	
H36C	0.5927	−0.1797	0.7390	0.046*	
C37	0.0993 (10)	−0.0538 (10)	1.0041 (7)	0.027 (2)	
H37A	0.0776	−0.1431	1.0152	0.040*	
H37B	0.0186	−0.0505	0.9520	0.040*	
H37C	0.1162	0.0211	1.0766	0.040*	
C38	0.4313 (8)	0.3543 (8)	0.8048 (6)	0.0110 (14)	
C39	0.4676 (8)	0.3908 (8)	0.7070 (6)	0.0116 (13)	
C40	0.5570 (8)	0.3281 (8)	0.6450 (6)	0.0164 (15)	
H40	0.5960	0.2631	0.6669	0.020*	
C41	0.5898 (8)	0.3590 (8)	0.5526 (6)	0.0195 (15)	
H41	0.6546	0.3190	0.5136	0.023*	
C42	0.5275 (9)	0.4492 (9)	0.5163 (6)	0.031 (2)	
H42	0.5463	0.4670	0.4505	0.037*	
C43	0.4391 (8)	0.5120 (7)	0.5758 (6)	0.0229 (16)	
H43	0.3980	0.5742	0.5513	0.028*	
C44	0.4092 (8)	0.4853 (8)	0.6722 (6)	0.0178 (15)	
H44	0.3497	0.5308	0.7139	0.021*	
C45	0.4363 (8)	0.4568 (8)	0.9050 (6)	0.0129 (13)	
C46	0.4133 (8)	0.4318 (7)	1.0041 (6)	0.0145 (15)	
C47	0.4402 (6)	0.5739 (5)	1.0964 (4)	0.0146 (10)	
C48	0.3087 (6)	0.6237 (5)	1.0558 (4)	0.0187 (10)	
H48A	0.2185	0.5495	1.0360	0.022*	
H48B	0.3013	0.7066	1.1153	0.022*	
C49	0.3407 (6)	0.6578 (5)	0.9512 (4)	0.0197 (10)	
H49A	0.3569	0.7579	0.9648	0.024*	
H49B	0.2622	0.6045	0.8844	0.024*	
C50	0.4792 (6)	0.6123 (5)	0.9367 (4)	0.0146 (10)	
H50	0.5329	0.6475	0.8856	0.018*	
C51	0.5615 (6)	0.6616 (5)	1.0609 (4)	0.0139 (9)	
C52	0.4574 (9)	0.5748 (9)	1.2163 (6)	0.0194 (17)	
H52A	0.5369	0.5370	1.2330	0.029*	
H52B	0.3694	0.5180	1.2247	0.029*	
H52C	0.4771	0.6700	1.2687	0.029*	
C53	0.5972 (8)	0.8174 (8)	1.1258 (6)	0.0191 (15)	
H53A	0.6490	0.8386	1.2028	0.029*	
H53B	0.5087	0.8456	1.1281	0.029*	
H53C	0.6566	0.8678	1.0879	0.029*	
C54	0.7017 (6)	0.6204 (5)	1.0730 (5)	0.0189 (10)	
H54A	0.6832	0.5210	1.0321	0.028*	
H54B	0.7414	0.6415	1.1529	0.028*	
H54C	0.7700	0.6724	1.0417	0.028*	

### Atomic displacement parameters (Å^2^)

**Table:**

	*U*^11^	*U*^22^	*U*^33^	*U*^12^	*U*^13^	*U*^23^
Ru1	0.0129 (3)	0.0073 (2)	0.0088 (2)	0.00148 (19)	0.00161 (18)	0.00126 (19)
Cl1	0.0181 (9)	0.0182 (10)	0.0176 (9)	0.0081 (8)	0.0042 (7)	0.0075 (8)
O1	0.019 (3)	0.011 (3)	0.007 (2)	0.005 (2)	0.005 (2)	0.002 (2)
O2	0.030 (3)	0.006 (3)	0.009 (2)	0.002 (2)	0.002 (2)	0.000 (2)
C1	0.020 (3)	0.014 (3)	0.019 (3)	0.010 (2)	0.007 (2)	0.008 (2)
C2	0.026 (3)	0.017 (3)	0.008 (2)	0.008 (2)	0.0018 (19)	0.006 (2)
C3	0.026 (3)	0.017 (3)	0.008 (2)	0.008 (2)	0.0018 (19)	0.006 (2)
C4	0.020 (3)	0.014 (3)	0.019 (3)	0.010 (2)	0.007 (2)	0.008 (2)
C5	0.017 (3)	0.010 (3)	0.021 (3)	0.000 (2)	0.003 (3)	0.005 (3)
C6	0.018 (3)	0.012 (3)	0.009 (3)	0.007 (2)	0.008 (2)	0.002 (2)
C7	0.011 (4)	0.019 (4)	0.022 (4)	0.005 (3)	−0.001 (3)	0.008 (3)
C8	0.044 (5)	0.015 (4)	0.043 (5)	0.013 (4)	0.018 (4)	0.007 (4)
C9	0.008 (3)	0.020 (4)	0.037 (4)	−0.004 (3)	0.002 (3)	0.008 (4)
C10	0.022 (4)	0.018 (4)	0.032 (5)	0.002 (3)	0.014 (3)	0.009 (4)
C11	0.013 (3)	0.013 (4)	0.018 (3)	0.006 (3)	0.000 (3)	0.007 (3)
C12	0.019 (3)	0.011 (3)	0.013 (3)	−0.001 (2)	−0.001 (2)	0.005 (2)
C13	0.022 (4)	0.014 (3)	0.013 (3)	0.006 (2)	0.000 (2)	0.001 (2)
C14	0.029 (4)	0.020 (3)	0.018 (3)	−0.001 (3)	−0.005 (3)	0.009 (2)
C15	0.029 (4)	0.020 (3)	0.012 (3)	−0.006 (3)	0.009 (2)	0.008 (2)
C16	0.032 (4)	0.019 (3)	0.018 (3)	0.005 (3)	0.005 (3)	0.002 (3)
C17	0.016 (3)	0.014 (3)	0.013 (3)	0.001 (2)	0.002 (2)	0.004 (2)
C18	0.020 (3)	0.006 (3)	0.013 (3)	0.002 (2)	0.000 (2)	0.002 (2)
C19	0.013 (3)	0.017 (3)	0.008 (3)	0.004 (2)	0.006 (2)	0.002 (2)
C20	0.021 (3)	0.011 (2)	0.014 (2)	0.004 (2)	0.002 (2)	0.0004 (18)
C21	0.021 (3)	0.011 (2)	0.018 (2)	0.005 (2)	0.002 (2)	−0.0015 (18)
C22	0.018 (3)	0.014 (2)	0.014 (2)	−0.005 (2)	0.002 (2)	0.0034 (18)
C23	0.020 (3)	0.016 (2)	0.018 (2)	0.0001 (19)	0.007 (2)	0.003 (2)
C24	0.010 (2)	0.015 (2)	0.021 (2)	0.0031 (18)	−0.0017 (19)	0.001 (2)
C25	0.026 (5)	0.019 (4)	0.010 (3)	0.000 (3)	0.001 (3)	−0.008 (3)
C26	0.027 (4)	0.010 (3)	0.022 (3)	0.005 (3)	−0.007 (3)	−0.004 (2)
C27	0.016 (3)	0.021 (3)	0.032 (3)	0.009 (2)	−0.004 (2)	0.001 (2)
Ru2	0.0126 (2)	0.0070 (2)	0.0082 (2)	0.00274 (19)	0.00164 (18)	0.00139 (18)
Cl2	0.0167 (9)	0.0215 (10)	0.0159 (9)	0.0108 (8)	0.0041 (7)	0.0048 (8)
O3	0.012 (3)	0.009 (3)	0.016 (3)	0.001 (2)	0.001 (2)	0.004 (2)
O4	0.013 (2)	0.011 (3)	0.010 (2)	0.002 (2)	0.0063 (19)	0.002 (2)
C28	0.012 (3)	0.009 (3)	0.015 (3)	0.003 (2)	0.006 (2)	0.004 (2)
C29	0.011 (3)	0.009 (3)	0.018 (3)	0.002 (2)	−0.002 (2)	0.002 (2)
C30	0.015 (3)	0.009 (3)	0.017 (3)	0.001 (2)	0.007 (2)	0.006 (2)
C31	0.012 (3)	0.009 (3)	0.015 (3)	0.003 (2)	0.006 (2)	0.004 (2)
C32	0.017 (3)	0.004 (2)	0.014 (3)	0.002 (2)	0.000 (2)	0.0004 (19)
C33	0.017 (3)	0.004 (2)	0.014 (3)	0.002 (2)	0.000 (2)	0.0004 (19)
C34	0.017 (4)	0.019 (4)	0.017 (4)	0.006 (3)	0.014 (3)	0.004 (3)
C35	0.026 (5)	0.028 (5)	0.029 (4)	0.007 (4)	0.004 (3)	0.003 (4)
C36	0.019 (4)	0.040 (5)	0.044 (5)	0.020 (4)	0.017 (4)	0.018 (4)
C37	0.038 (5)	0.025 (5)	0.024 (4)	0.011 (4)	0.016 (4)	0.012 (4)
C38	0.010 (3)	0.013 (4)	0.009 (3)	0.001 (3)	0.004 (2)	0.003 (3)
C39	0.008 (3)	0.009 (3)	0.010 (3)	−0.002 (2)	−0.001 (2)	−0.003 (2)
C40	0.017 (3)	0.011 (3)	0.018 (3)	0.001 (3)	0.003 (2)	0.004 (3)
C41	0.012 (3)	0.021 (4)	0.019 (3)	−0.004 (2)	0.005 (2)	0.003 (3)
C42	0.050 (5)	0.025 (4)	0.015 (3)	0.010 (3)	0.000 (3)	0.009 (3)
C43	0.035 (4)	0.017 (3)	0.013 (3)	0.004 (3)	−0.006 (3)	0.007 (2)
C44	0.018 (3)	0.017 (3)	0.018 (3)	0.004 (2)	0.003 (2)	0.007 (2)
C45	0.017 (3)	0.009 (3)	0.012 (3)	0.001 (2)	0.002 (2)	0.004 (2)
C46	0.016 (3)	0.005 (3)	0.017 (3)	0.001 (2)	−0.005 (2)	0.001 (2)
C47	0.023 (3)	0.006 (2)	0.012 (2)	0.002 (2)	0.004 (2)	−0.0003 (16)
C48	0.021 (3)	0.015 (3)	0.019 (2)	0.008 (2)	0.004 (2)	0.0034 (19)
C49	0.024 (3)	0.010 (2)	0.022 (2)	0.006 (2)	−0.004 (2)	0.0028 (19)
C50	0.020 (3)	0.007 (2)	0.011 (2)	−0.001 (2)	−0.001 (2)	−0.0001 (16)
C51	0.015 (3)	0.008 (2)	0.015 (2)	0.0006 (18)	0.0023 (18)	0.0020 (17)
C52	0.025 (4)	0.016 (4)	0.016 (4)	0.002 (3)	0.007 (3)	0.005 (3)
C53	0.021 (3)	0.014 (3)	0.021 (3)	0.002 (2)	0.007 (2)	0.006 (2)
C54	0.017 (3)	0.018 (3)	0.022 (3)	0.002 (2)	0.005 (2)	0.008 (2)

### Geometric parameters (Å, °)

**Table:**

Ru1—O2	2.077 (5)	Ru2—O3	2.073 (5)
Ru1—O1	2.084 (5)	Ru2—O4	2.086 (5)
Ru1—C3	2.151 (7)	Ru2—C30	2.141 (7)
Ru1—C2	2.160 (7)	Ru2—C29	2.160 (7)
Ru1—C5	2.163 (8)	Ru2—C32	2.168 (8)
Ru1—C6	2.170 (8)	Ru2—C31	2.186 (8)
Ru1—C4	2.188 (9)	Ru2—C28	2.190 (7)
Ru1—C1	2.200 (8)	Ru2—C33	2.198 (8)
Ru1—Cl1	2.4069 (19)	Ru2—Cl2	2.4108 (18)
O1—C11	1.293 (10)	O3—C38	1.293 (9)
O2—C19	1.246 (9)	O4—C46	1.259 (9)
C1—C6	1.438 (12)	C28—C29	1.397 (10)
C1—C2	1.439 (10)	C28—C33	1.442 (11)
C1—C7	1.483 (12)	C28—C34	1.513 (10)
C2—C3	1.385 (12)	C29—C30	1.461 (11)
C2—H2	0.9500	C29—H29	0.9500
C3—C4	1.437 (12)	C30—C31	1.384 (11)
C3—H3	0.9500	C30—H30	0.9500
C4—C5	1.436 (11)	C31—C32	1.428 (10)
C4—C10	1.482 (11)	C31—C37	1.506 (11)
C5—C6	1.363 (11)	C32—C33	1.424 (11)
C5—H5	0.9500	C32—H32	0.9500
C6—H6	0.9500	C33—H33	0.9500
C7—C9	1.541 (12)	C34—C36	1.517 (11)
C7—C8	1.568 (11)	C34—C35	1.516 (13)
C7—H7	1.0000	C34—H34	1.0000
C8—H8A	0.9800	C35—H35A	0.9800
C8—H8B	0.9800	C35—H35B	0.9800
C8—H8C	0.9800	C35—H35C	0.9800
C9—H9A	0.9800	C36—H36A	0.9800
C9—H9B	0.9800	C36—H36B	0.9800
C9—H9C	0.9800	C36—H36C	0.9800
C10—H10A	0.9800	C37—H37A	0.9800
C10—H10B	0.9800	C37—H37B	0.9800
C10—H10C	0.9800	C37—H37C	0.9800
C11—C18	1.384 (11)	C38—C45	1.383 (10)
C11—C12	1.510 (11)	C38—C39	1.482 (10)
C12—C17	1.379 (11)	C39—C40	1.397 (10)
C12—C13	1.392 (10)	C39—C44	1.409 (10)
C13—C14	1.376 (10)	C40—C41	1.379 (11)
C13—H13	0.9500	C40—H40	0.9500
C14—C15	1.386 (11)	C41—C42	1.397 (11)
C14—H14	0.9500	C41—H41	0.9500
C15—C16	1.381 (11)	C42—C43	1.373 (12)
C15—H15	0.9500	C42—H42	0.9500
C16—C17	1.405 (11)	C43—C44	1.401 (10)
C16—H16	0.9500	C43—H43	0.9500
C17—H17	0.9500	C44—H44	0.9500
C18—C19	1.429 (10)	C45—C46	1.409 (11)
C18—C22	1.544 (9)	C45—C50	1.511 (9)
C19—C20	1.515 (9)	C46—C47	1.536 (8)
C20—C25	1.512 (9)	C47—C52	1.516 (9)
C20—C24	1.553 (7)	C47—C51	1.553 (7)
C20—C21	1.554 (7)	C47—C48	1.583 (7)
C21—C27	1.533 (8)	C48—C49	1.543 (7)
C21—C26	1.536 (9)	C48—H48A	0.9900
C21—C22	1.559 (7)	C48—H48B	0.9900
C22—C23	1.530 (8)	C49—C50	1.555 (7)
C22—H22	1.0000	C49—H49A	0.9900
C23—C24	1.551 (7)	C49—H49B	0.9900
C23—H23A	0.9900	C50—C51	1.553 (6)
C23—H23B	0.9900	C50—H50	1.0000
C24—H24A	0.9900	C51—C53	1.532 (9)
C24—H24B	0.9900	C51—C54	1.536 (7)
C25—H25A	0.9800	C52—H52A	0.9800
C25—H25B	0.9800	C52—H52B	0.9800
C25—H25C	0.9800	C52—H52C	0.9800
C26—H26A	0.9800	C53—H53A	0.9800
C26—H26B	0.9800	C53—H53B	0.9800
C26—H26C	0.9800	C53—H53C	0.9800
C27—H27A	0.9800	C54—H54A	0.9800
C27—H27B	0.9800	C54—H54B	0.9800
C27—H27C	0.9800	C54—H54C	0.9800
			
O2—Ru1—O1	89.7 (2)	O3—Ru2—O4	89.7 (2)
O2—Ru1—C3	87.5 (3)	O3—Ru2—C30	151.5 (2)
O1—Ru1—C3	152.5 (3)	O4—Ru2—C30	89.0 (3)
O2—Ru1—C2	91.9 (3)	O3—Ru2—C29	112.2 (3)
O1—Ru1—C2	115.4 (3)	O4—Ru2—C29	95.0 (3)
C3—Ru1—C2	37.5 (3)	C30—Ru2—C29	39.7 (3)
O2—Ru1—C5	149.6 (3)	O3—Ru2—C32	121.0 (2)
O1—Ru1—C5	120.1 (3)	O4—Ru2—C32	148.3 (2)
C3—Ru1—C5	68.9 (3)	C30—Ru2—C32	68.0 (3)
C2—Ru1—C5	80.9 (3)	C29—Ru2—C32	81.3 (3)
O2—Ru1—C6	159.5 (2)	O3—Ru2—C31	159.2 (2)
O1—Ru1—C6	93.3 (2)	O4—Ru2—C31	110.9 (2)
C3—Ru1—C6	80.5 (3)	C30—Ru2—C31	37.3 (3)
C2—Ru1—C6	68.5 (3)	C29—Ru2—C31	69.8 (3)
C5—Ru1—C6	36.7 (3)	C32—Ru2—C31	38.3 (3)
O2—Ru1—C4	111.4 (3)	O3—Ru2—C28	87.6 (2)
O1—Ru1—C4	158.6 (2)	O4—Ru2—C28	124.1 (3)
C3—Ru1—C4	38.7 (3)	C30—Ru2—C28	69.8 (3)
C2—Ru1—C4	69.0 (3)	C29—Ru2—C28	37.5 (3)
C5—Ru1—C4	38.5 (3)	C32—Ru2—C28	69.4 (3)
C6—Ru1—C4	68.2 (3)	C31—Ru2—C28	82.8 (3)
O2—Ru1—C1	121.5 (3)	O3—Ru2—C33	91.9 (3)
O1—Ru1—C1	89.5 (3)	O4—Ru2—C33	162.2 (2)
C3—Ru1—C1	69.0 (3)	C30—Ru2—C33	81.2 (3)
C2—Ru1—C1	38.5 (3)	C29—Ru2—C33	68.1 (3)
C5—Ru1—C1	68.6 (3)	C32—Ru2—C33	38.1 (3)
C6—Ru1—C1	38.4 (3)	C31—Ru2—C33	69.2 (3)
C4—Ru1—C1	82.6 (3)	C28—Ru2—C33	38.4 (3)
O2—Ru1—Cl1	85.99 (17)	O3—Ru2—Cl2	86.42 (15)
O1—Ru1—Cl1	86.05 (15)	O4—Ru2—Cl2	84.06 (16)
C3—Ru1—Cl1	121.0 (2)	C30—Ru2—Cl2	121.7 (2)
C2—Ru1—Cl1	158.5 (2)	C29—Ru2—Cl2	161.4 (2)
C5—Ru1—Cl1	90.1 (2)	C32—Ru2—Cl2	89.8 (2)
C6—Ru1—Cl1	114.4 (2)	C31—Ru2—Cl2	93.18 (19)
C4—Ru1—Cl1	91.8 (2)	C28—Ru2—Cl2	151.1 (2)
C1—Ru1—Cl1	152.2 (2)	C33—Ru2—Cl2	113.7 (2)
C11—O1—Ru1	126.1 (5)	C38—O3—Ru2	126.7 (5)
C19—O2—Ru1	124.1 (5)	C46—O4—Ru2	121.8 (5)
C6—C1—C2	115.7 (7)	C29—C28—C33	118.5 (7)
C6—C1—C7	121.4 (7)	C29—C28—C34	123.1 (7)
C2—C1—C7	122.8 (8)	C33—C28—C34	118.4 (7)
C6—C1—Ru1	69.7 (4)	C29—C28—Ru2	70.1 (4)
C2—C1—Ru1	69.2 (4)	C33—C28—Ru2	71.1 (4)
C7—C1—Ru1	127.8 (5)	C34—C28—Ru2	130.4 (5)
C3—C2—C1	121.7 (8)	C28—C29—C30	120.3 (7)
C3—C2—Ru1	70.9 (4)	C28—C29—Ru2	72.4 (4)
C1—C2—Ru1	72.2 (4)	C30—C29—Ru2	69.4 (4)
C3—C2—H2	119.1	C28—C29—H29	119.9
C1—C2—H2	119.1	C30—C29—H29	119.9
Ru1—C2—H2	130.4	Ru2—C29—H29	131.0
C2—C3—C4	121.7 (7)	C31—C30—C29	121.7 (7)
C2—C3—Ru1	71.6 (4)	C31—C30—Ru2	73.1 (4)
C4—C3—Ru1	72.0 (4)	C29—C30—Ru2	70.8 (4)
C2—C3—H3	119.2	C31—C30—H30	119.1
C4—C3—H3	119.2	C29—C30—H30	119.1
Ru1—C3—H3	129.8	Ru2—C30—H30	129.4
C3—C4—C5	116.4 (7)	C30—C31—C32	117.9 (7)
C3—C4—C10	121.8 (8)	C30—C31—C37	121.6 (7)
C5—C4—C10	121.7 (8)	C32—C31—C37	120.4 (7)
C3—C4—Ru1	69.3 (5)	C30—C31—Ru2	69.6 (5)
C5—C4—Ru1	69.8 (4)	C32—C31—Ru2	70.2 (4)
C10—C4—Ru1	128.6 (6)	C37—C31—Ru2	128.5 (5)
C6—C5—C4	121.7 (8)	C33—C32—C31	121.7 (7)
C6—C5—Ru1	71.9 (5)	C33—C32—Ru2	72.1 (4)
C4—C5—Ru1	71.7 (5)	C31—C32—Ru2	71.5 (4)
C6—C5—H5	119.2	C33—C32—H32	119.2
C4—C5—H5	119.2	C31—C32—H32	119.2
Ru1—C5—H5	129.8	Ru2—C32—H32	129.8
C5—C6—C1	122.8 (7)	C32—C33—C28	119.9 (7)
C5—C6—Ru1	71.4 (5)	C32—C33—Ru2	69.8 (4)
C1—C6—Ru1	71.9 (4)	C28—C33—Ru2	70.5 (4)
C5—C6—H6	118.6	C32—C33—H33	120.0
C1—C6—H6	118.6	C28—C33—H33	120.0
Ru1—C6—H6	131.1	Ru2—C33—H33	132.6
C1—C7—C9	116.4 (7)	C28—C34—C36	108.0 (7)
C1—C7—C8	109.0 (7)	C28—C34—C35	111.9 (7)
C9—C7—C8	108.2 (7)	C36—C34—C35	113.4 (7)
C1—C7—H7	107.7	C28—C34—H34	107.8
C9—C7—H7	107.7	C36—C34—H34	107.8
C8—C7—H7	107.7	C35—C34—H34	107.8
C7—C8—H8A	109.5	C34—C35—H35A	109.5
C7—C8—H8B	109.5	C34—C35—H35B	109.5
H8A—C8—H8B	109.5	H35A—C35—H35B	109.5
C7—C8—H8C	109.5	C34—C35—H35C	109.5
H8A—C8—H8C	109.5	H35A—C35—H35C	109.5
H8B—C8—H8C	109.5	H35B—C35—H35C	109.5
C7—C9—H9A	109.5	C34—C36—H36A	109.5
C7—C9—H9B	109.5	C34—C36—H36B	109.5
H9A—C9—H9B	109.5	H36A—C36—H36B	109.5
C7—C9—H9C	109.5	C34—C36—H36C	109.5
H9A—C9—H9C	109.5	H36A—C36—H36C	109.5
H9B—C9—H9C	109.5	H36B—C36—H36C	109.5
C4—C10—H10A	109.5	C31—C37—H37A	109.5
C4—C10—H10B	109.5	C31—C37—H37B	109.5
H10A—C10—H10B	109.5	H37A—C37—H37B	109.5
C4—C10—H10C	109.5	C31—C37—H37C	109.5
H10A—C10—H10C	109.5	H37A—C37—H37C	109.5
H10B—C10—H10C	109.5	H37B—C37—H37C	109.5
O1—C11—C18	125.8 (7)	O3—C38—C45	125.3 (7)
O1—C11—C12	113.7 (7)	O3—C38—C39	114.3 (7)
C18—C11—C12	120.4 (7)	C45—C38—C39	120.5 (7)
C17—C12—C13	119.8 (7)	C40—C39—C44	118.6 (7)
C17—C12—C11	119.4 (7)	C40—C39—C38	120.4 (7)
C13—C12—C11	120.7 (7)	C44—C39—C38	121.0 (7)
C14—C13—C12	120.8 (7)	C41—C40—C39	121.1 (7)
C14—C13—H13	119.6	C41—C40—H40	119.4
C12—C13—H13	119.6	C39—C40—H40	119.4
C13—C14—C15	119.3 (7)	C40—C41—C42	119.9 (8)
C13—C14—H14	120.3	C40—C41—H41	120.1
C15—C14—H14	120.3	C42—C41—H41	120.1
C16—C15—C14	120.9 (7)	C43—C42—C41	120.0 (7)
C16—C15—H15	119.5	C43—C42—H42	120.0
C14—C15—H15	119.5	C41—C42—H42	120.0
C15—C16—C17	119.3 (8)	C42—C43—C44	120.6 (7)
C15—C16—H16	120.4	C42—C43—H43	119.7
C17—C16—H16	120.4	C44—C43—H43	119.7
C12—C17—C16	119.9 (7)	C43—C44—C39	119.7 (7)
C12—C17—H17	120.1	C43—C44—H44	120.1
C16—C17—H17	120.1	C39—C44—H44	120.1
C11—C18—C19	124.6 (7)	C38—C45—C46	124.0 (7)
C11—C18—C22	128.8 (7)	C38—C45—C50	129.8 (6)
C19—C18—C22	105.0 (6)	C46—C45—C50	106.0 (6)
O2—C19—C18	129.6 (7)	O4—C46—C45	131.7 (7)
O2—C19—C20	122.9 (6)	O4—C46—C47	121.5 (6)
C18—C19—C20	106.8 (6)	C45—C46—C47	106.7 (6)
C25—C20—C19	113.9 (5)	C52—C47—C46	115.8 (5)
C25—C20—C24	116.1 (5)	C52—C47—C51	120.5 (5)
C19—C20—C24	103.3 (4)	C46—C47—C51	100.4 (4)
C25—C20—C21	118.6 (5)	C52—C47—C48	114.6 (5)
C19—C20—C21	100.8 (4)	C46—C47—C48	101.8 (4)
C24—C20—C21	101.8 (4)	C51—C47—C48	100.9 (4)
C27—C21—C26	108.7 (5)	C49—C48—C47	104.5 (4)
C27—C21—C20	113.4 (5)	C49—C48—H48A	110.8
C26—C21—C20	114.1 (5)	C47—C48—H48A	110.8
C27—C21—C22	113.9 (4)	C49—C48—H48B	110.8
C26—C21—C22	112.8 (5)	C47—C48—H48B	110.8
C20—C21—C22	93.4 (4)	H48A—C48—H48B	108.9
C23—C22—C18	105.5 (4)	C48—C49—C50	102.0 (4)
C23—C22—C21	102.6 (4)	C48—C49—H49A	111.4
C18—C22—C21	100.6 (4)	C50—C49—H49A	111.4
C23—C22—H22	115.4	C48—C49—H49B	111.4
C18—C22—H22	115.4	C50—C49—H49B	111.4
C21—C22—H22	115.4	H49A—C49—H49B	109.2
C22—C23—C24	102.5 (4)	C45—C50—C51	102.2 (4)
C22—C23—H23A	111.3	C45—C50—C49	105.8 (5)
C24—C23—H23A	111.3	C51—C50—C49	101.7 (4)
C22—C23—H23B	111.3	C45—C50—H50	115.2
C24—C23—H23B	111.3	C51—C50—H50	115.2
H23A—C23—H23B	109.2	C49—C50—H50	115.2
C23—C24—C20	104.3 (4)	C53—C51—C54	107.1 (5)
C23—C24—H24A	110.9	C53—C51—C47	113.7 (5)
C20—C24—H24A	110.9	C54—C51—C47	113.7 (4)
C23—C24—H24B	110.9	C53—C51—C50	115.3 (4)
C20—C24—H24B	110.9	C54—C51—C50	113.2 (4)
H24A—C24—H24B	108.9	C47—C51—C50	93.8 (4)
C20—C25—H25A	109.5	C47—C52—H52A	109.5
C20—C25—H25B	109.5	C47—C52—H52B	109.5
H25A—C25—H25B	109.5	H52A—C52—H52B	109.5
C20—C25—H25C	109.5	C47—C52—H52C	109.5
H25A—C25—H25C	109.5	H52A—C52—H52C	109.5
H25B—C25—H25C	109.5	H52B—C52—H52C	109.5
C21—C26—H26A	109.5	C51—C53—H53A	109.5
C21—C26—H26B	109.5	C51—C53—H53B	109.5
H26A—C26—H26B	109.5	H53A—C53—H53B	109.5
C21—C26—H26C	109.5	C51—C53—H53C	109.5
H26A—C26—H26C	109.5	H53A—C53—H53C	109.5
H26B—C26—H26C	109.5	H53B—C53—H53C	109.5
C21—C27—H27A	109.5	C51—C54—H54A	109.5
C21—C27—H27B	109.5	C51—C54—H54B	109.5
H27A—C27—H27B	109.5	H54A—C54—H54B	109.5
C21—C27—H27C	109.5	C51—C54—H54C	109.5
H27A—C27—H27C	109.5	H54A—C54—H54C	109.5
H27B—C27—H27C	109.5	H54B—C54—H54C	109.5

### Hydrogen-bond geometry (Å, °)

**Table:**

*D*—H···*A*	*D*—H	H···*A*	*D*···*A*	*D*—H···*A*
C43—H43···Cl1	0.95	2.69	3.605 (7)	162
C14—H14···Cl2	0.95	2.78	3.731 (7)	178
